# The quantification and potential impact of dark current on treatments with an MR‐guided radiotherapy (MRgRT) system

**DOI:** 10.1002/acm2.13059

**Published:** 2020-10-29

**Authors:** Andrew J. Shepard, Kathryn E. Mittauer, John E. Bayouth, Poonam Yadav

**Affiliations:** ^1^ Department of Human Oncology School of Medicine and Public Health University of Wisconsin‐Madison Madison WI USA; ^2^ Present address: Department of Radiation Oncology University of Iowa Iowa City IA 52242 USA; ^3^ Present address: Department of Radiation Oncology Miami Cancer Institute Baptist Health South Florida Miami FL 33176 USA

**Keywords:** dark current, gating, MR‐guided radiotherapy, beam‐hold

## Abstract

**Purpose:**

Dark current radiation produced during linac beam‐hold has the potential to lead to unplanned dose delivered to the patient. With the increased usage of motion management and step‐and‐shoot IMRT deliveries for MR‐guided systems leading to increased beam‐hold time, it is necessary to consider the impact of dark current radiation on patient treatments.

**Methods:**

The relative dose rate due to dark current for the ViewRay MRIdian linac was measured longitudinally over 15 months (June 2018‐August 2019). Ion chamber measurements were acquired with the linac in the beam‐hold state and the beam‐on state, with the ratio representing the relative dark current dose rate. The potential contribution of the dark current dose to the overall prescription was retrospectively analyzed for 972 fractions from 83 patients over the same time period. The amount of time spent in the beam‐hold state was combined with the monthly measured relative dark current dose rate to estimate the dark current dose contribution.

**Results:**

The relative dark current dose rate compared to the beam‐on dose rate was 0.12% ± 0.027%. In a near worst‐case estimation, the dark current dose contribution accounted for 0.90% ± 0.67% of the prescription dose across all fractions (3.61% maximum). Gantry and MLC motion between segments accounted for 87% of the dark current contribution, with the remaining 13% attributable to gating during segment delivery. The largest dark current contributions were associated with plans delivering a small dose per treatment segment.

**Conclusions:**

The dark current associated with new clinical treatment units should be considered prior to treatment delivery to ensure it will not lead to dosimetric inaccuracies. For the MRIdian linac system investigated in this work, the contribution from dark current remained relatively low, though users should be cognizant of the larger potential dosimetric contribution for plans with small doses per segment.

## 1 INTRODUCTION

With the increased usage of gating within radiotherapy, and the return to step‐and‐shoot deliveries for new treatment delivery systems, there can be an extended amount of time spent in the linac beam‐hold state throughout the duration of a treatment. The linac is placed in the beam‐hold state during gating events, and as the gantry and multileaf collimator (MLC) move into position between segments of a step‐and‐shoot delivery. This is particularly relevant for systems such as the ViewRay MRIdian (ViewRay Inc., Cleveland, OH) linac which necessitate the use of step‐and‐shoot deliveries for intensity modulated radiotherapy (IMRT) treatments.^1^ Increased time in the beam‐hold state not only increases the overall time the patient is on the table, but also may subject the patient to dark current radiation, leading to an increased total body dose and a decrease in delivery accuracy.

The linac beam‐hold state is meant to facilitate a rapid response when a beam‐on event is desired. To achieve this, many of the components required for beam generation remain on, just in a reduced or asynchronous state. During beam‐hold, the electron gun and the radio frequency (RF) wave are out of sync so as not to produce a primary beam; however, due to the presence of the RF wave, there is still the possibility for electrons along the walls of the cavity to be accelerated down the wave guide and for radiation to reach the patient.^2^, ^3^, ^4^ The radiation produced is known as dark current and can be the result of high current densities at surface irregularities of the cavity, or contaminants that remain on the surface of the cavity.^5^, ^6^

Cheng and Das initially investigated the contribution of dark current radiation and the effect of the initial pulse forming network (IPFN) setting on dark current suppression for a Siemens Primus machine.^3^, ^4^ The dark current radiation was measured to be 0.7% of the maximum dose for a 15 MV beam, but was reduced to an undetectable amount when the IPFN‐to‐PFN ratio was decreased to <0.8 (there was no dark current radiation detected for the 6 MV beam regardless of the setting).^3^, ^4^ Ultimately, the work suggested dark current measurements be a part of linac commissioning and lower energy photon beams be used for IMRT treatments.^4^ Additionally, work by Kim and Chang discussed the impact of dark current for the CyberKnife system, measuring a relative dose due to dark current of up to 0.6%.^7^ Outside of these works, to the authors’ knowledge there have been no further investigations published on linac dark current radiation in radiotherapy. This could be due to new delivery techniques (i.e., VMAT) reducing the amount of time spent in the beam‐hold state, or manufacturer implementation of improved dark current suppression; however, it is still prudent to investigate and verify the dark current contribution for all clinical linear accelerators. With the MRIdian linac system necessitating the use of step‐and‐shoot deliveries for IMRT, along with the increased prevalence of gated treatments, it is particularly important to verify that the dark current radiation present is at an acceptable level.

The MRIdian linac system performs step‐and‐shoot deliveries with real‐time MR image guidance to monitor and gate treatments based on target location throughout the fraction. With a significant amount of time spent moving the gantry (two minutes per rotation) and MLC (1.5‐1.8 cm per second for the MLC used in this work) between segments, as well as gating due to target motion, there is added interest in considering the dark current present. During installation, ViewRay ensures that the dark current radiation is limited to <0.1% of the nominal dose rate.^8^ Additionally, during treatment, a dark current dose rate interlock is employed that stops treatment if the average dark current dose rate exceeds 0.1% of the expected nominal dose rate.^9^ To reduce the dark current contribution and maintain it at an acceptable rate, ViewRay reduces the pulse repetition frequency (PRF) of the linac during the beam‐hold state to 5 Hz.

In this work, the magnitude of dark current radiation during the beam‐hold state was measured for the ViewRay MRIdian linac system and the potential impact that it may have on patient deliveries was investigated. The relative dark current dose rate was first assessed by comparing ion chamber measurements with the linac in the beam‐hold state to measurements with the linac in the beam‐on state for several months of the initial machine operation. Subsequently, a near worst‐case estimate of the potential contribution of dark current to patient deliveries was assessed by retrospectively analyzing the beam‐hold time from patient delivery reports and applying the measured dark current dose rate from the test scenario for the total beam‐hold time.

## 2 METHODS

### 2.1 Monthly monitoring of dark current

**Fig. 1:**
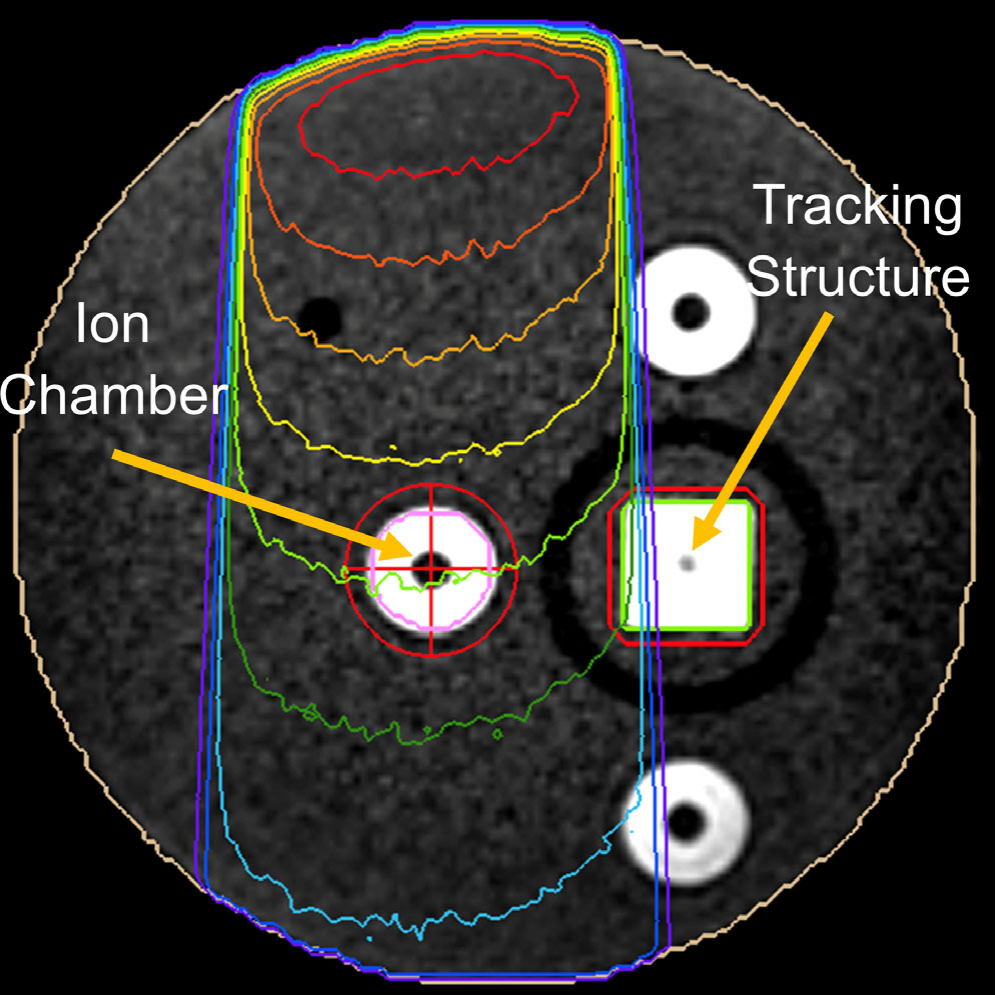
Phantom setup and dose distribution. The ion chamber was positioned within the center of an open field and remained stable for the duration of the measurement. The tracking structure had the ability to move in and out of the plane shown, with the beam‐hold state achieved by moving the tracking structure outside of the gating window.

The dose rate attributable to dark current for a ViewRay MRIdian linac was measured and compared to the beam‐on dose rate over the course of a 15‐month period from June 2018 to August 2019 (n = 9). In order to measure the dark current dose rate, an Exradin A28 ion chamber (Standard Imaging, Middleton, WI) was positioned lateral to a tracking structure (Fig. 1) within an MR‐compatible motion phantom (CIRS Inc., Norfolk, VA). A 3D MR image was acquired, and the phantom was registered to a reference MR image which was used for planning. The registration of the phantom ensured proper positioning of the ion chamber within the planned field. With the ion chamber positioned, a QA procedure consisting of a 10x10 cm^2^ equivalent field centered on the ion chamber with gating enabled was initiated. The system was set to gate based on the position of the tracking structure. The ion chamber was maintained at the center of the static 10x10 cm^2^ equivalent field and a 60 second measurement was taken with the beam in the hold state. Beam‐hold was attained by moving the tracking structure outside of the gating window. Note that while the tracking structure was moved from the gating window to initiate beam‐hold, the ion chamber did not move and was maintained at the center of the field. The 60 second measurement was not started until the linac was in the beam‐hold state.

Additionally, the measured readings were normalized to the beam‐on dose rate with a 60 second measurement acquired with the beam in the “on” state. The ion chamber was maintained in an identical position as was used for the measurement of the dark current; however, the tracking feature was within the gating window for the duration of the measurement and the beam operated as it would for treatment. The leakage of the ion chamber and electrometer used in this work was assessed at the initial implementation of the approach and was determined to be minimal, though it was not assessed at each measurement session.

### 2.2 Impact of dark current on patient deliveries

An IRB‐approved retrospective analysis of the potential effect of dark current radiation on the delivery of patient treatments was performed by analyzing patient delivery records. Utilizing the average dark current dose rate as determined from monthly measurements (as described in Section 2.1) and the known beam‐hold times associated with each treatment, the relative contribution due to dark current for each treatment was estimated. A MATLAB (R2018b, MathWorks, Torrance, CA) code was developed to extract gating times and the time between beam segments from patient delivery records. The total time that the linac was in the beam‐hold state was determined by combining the gating and inter‐segment times. The total beam‐hold time was then multiplied by the average dose rate due to dark current observed from monthly measurements to determine the dose from dark current in delivered patient plans. The total estimated dark current contribution was compared relative to the prescription dose for 972 fractions from 83 patients across 14 disease sites.

## 3 RESULTS

### 3.1 Monthly dark current quantification

**Fig. 2:**
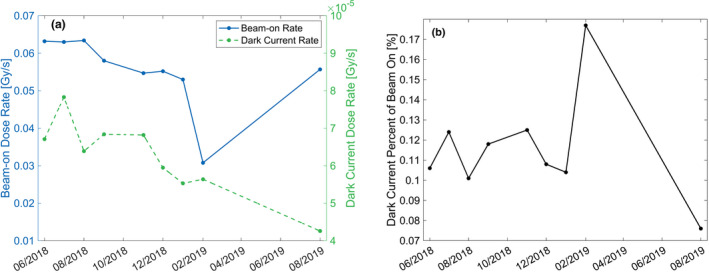
Monthly dark current dose rate. The monthly trend in the measured dose rate with the linac in the beam‐hold (dark current) and beam‐on state is provided in (a). The ratio of the dose rate during beam‐hold relative to beam‐on is provided in (b).

The average dose rate over the course of a 60‐second measurement was monitored on a near monthly basis with the linac in the beam‐on state and the beam‐hold state. The dose rate in the beam‐on and beam‐hold (dark current) state is shown in Fig. 2a over the course of the first 15 months of machine operation from June 2018 to August 2019. To monitor the change in the dark current dose rate over the course of time and account for changes in the beam‐on dose rate, the ratio of the dark current rate to the beam‐on rate was calculated and is provided in Fig. 2b.

The average dose rate due to dark current was 62.0 ± 10.3 μGy/s while the average dose rate with the linac in the beam‐on state was 5.52 ± 1.00 cGy/s for the ion chamber position analyzed. The ratio of the dark current dose rate relative to the beam‐on dose rate was 0.12% ± 0.027%. There was a consistent decrease in the beam‐on dose rate up until February 2019 as noted by daily QA measurements.^10^ A significant decrease in the beam‐on dose rate was observed during the February 2019 measurement and was likely the reason for the corresponding spike in relative dark current dose rate displayed in Fig. 2b. The decreased dose rate was communicated to the manufacturer and adjustments were made to return the dose rate to the expected level.

### 3.2 Impact on patient deliveries

The delivery reports from 83 patients and 972 total fractions were analyzed to determine the amount of treatment time spent in the beam‐hold state. The total time was further broken down into both beam gating due to target motion, as well as gantry and MLC movement. The average total beam‐hold time across all fractions was 687 ± 282 seconds, with 152 ± 190 seconds, and 534 ± 196 seconds due to gating and gantry/MLC movement, respectively. Utilizing the average dark current dose rate relative to the beam‐on dose rate observed during monthly QA, the total dose contribution due to dark current across all fractions was 0.90% ± 0.67% of the total prescription dose. The maximum fraction dose due to dark current was 3.61% (abdominal). Further breaking down the total contribution into the percentage attributable to gating and MLC/gantry motion, it was estimated that these components accounted for 0.12% ± 0.11%, and 0.78% ± 0.65% of the total prescription dose, respectively. Performing an identical analysis, but instead considering the average of all fractions for a given patient, the average contribution due to dark current per patient was 0.63% ± 0.56%, with a maximum contribution of 2.57%. Additionally, the contributions per patient attributable to gating and MLC/gantry motion were 0.13% ± 0.07% and 0.51% ± 0.55%, respectively.

**Fig. 3:**
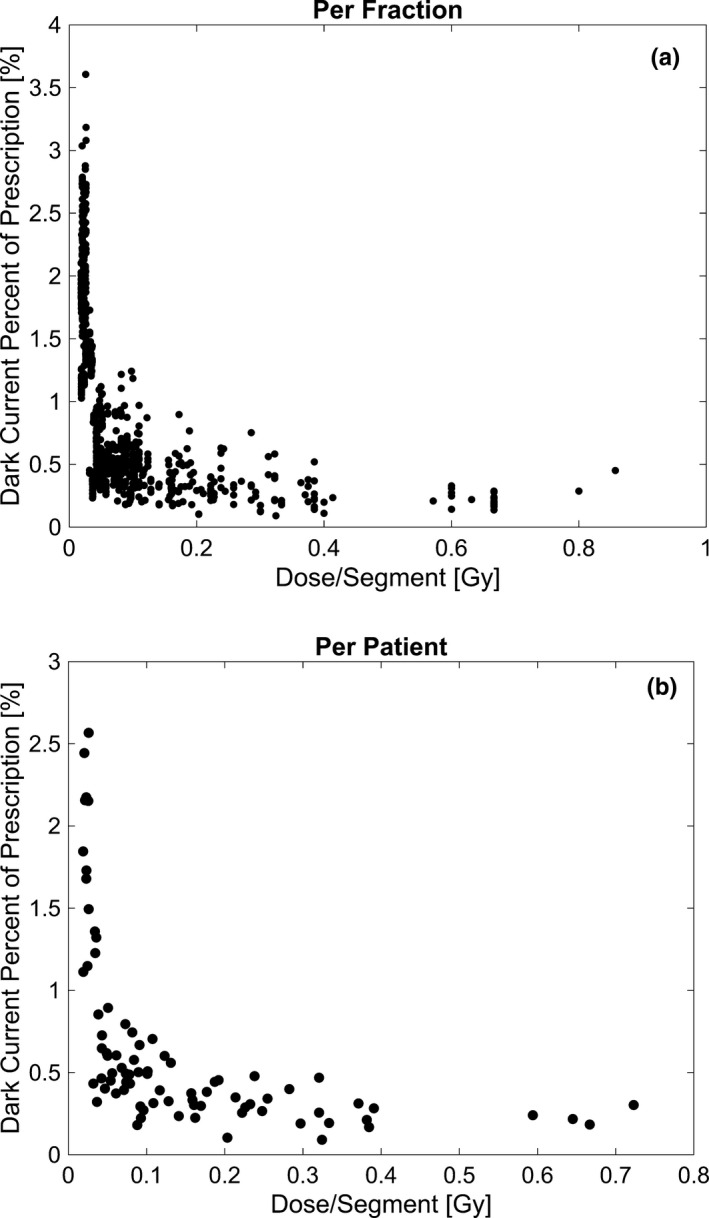
Dose per segment dependence. The dark current percentage contribution relative to the dose per segment is presented for the total (a) fractions and (b) patients analyzed.

**Fig. 4:**
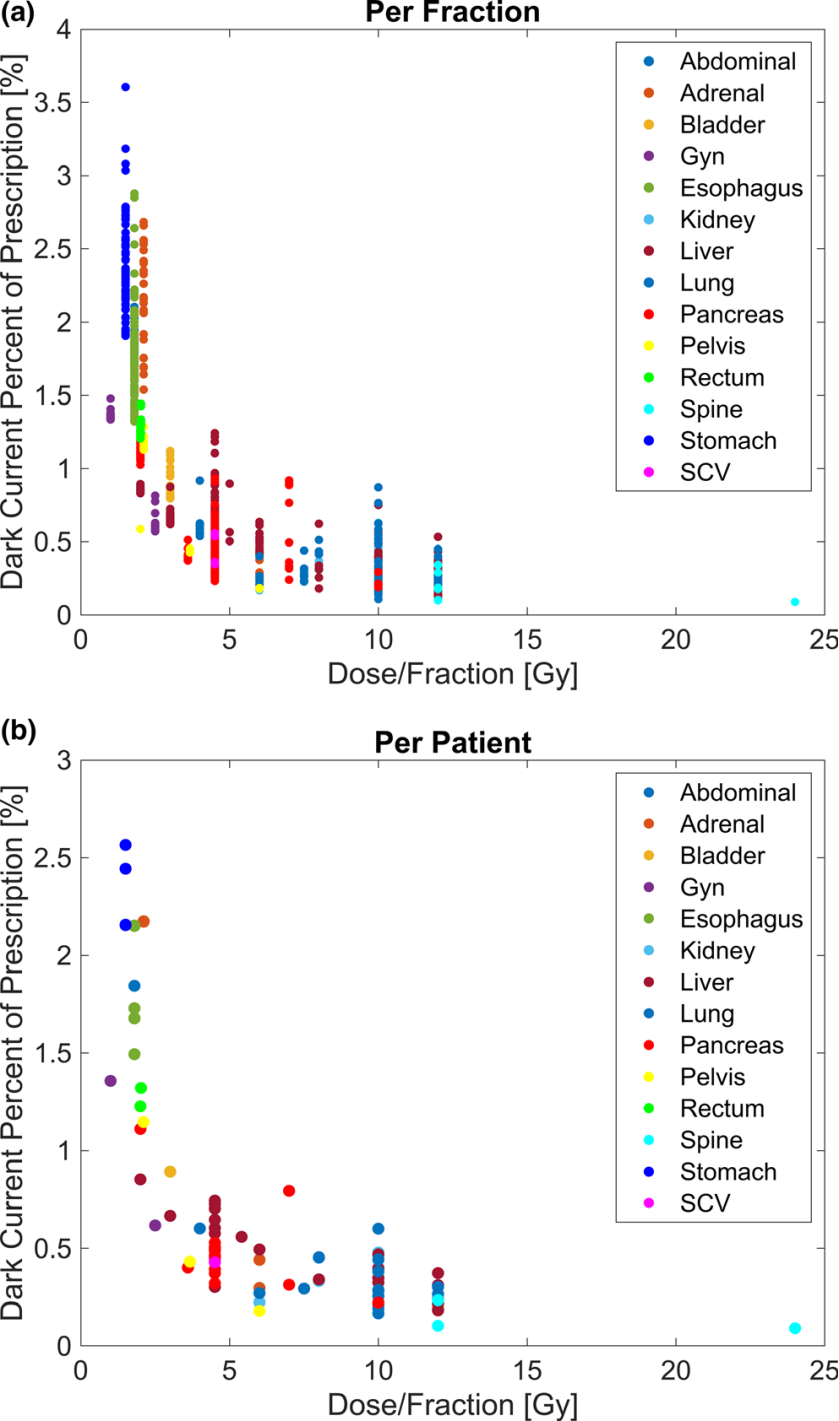
Dose per fraction dependence. The dark current percentage contribution relative to the dose per fraction for the total (a) fractions and (b) patients analyzed. Varying sites are indicated by different color scatter dots.

**Fig. 5:**
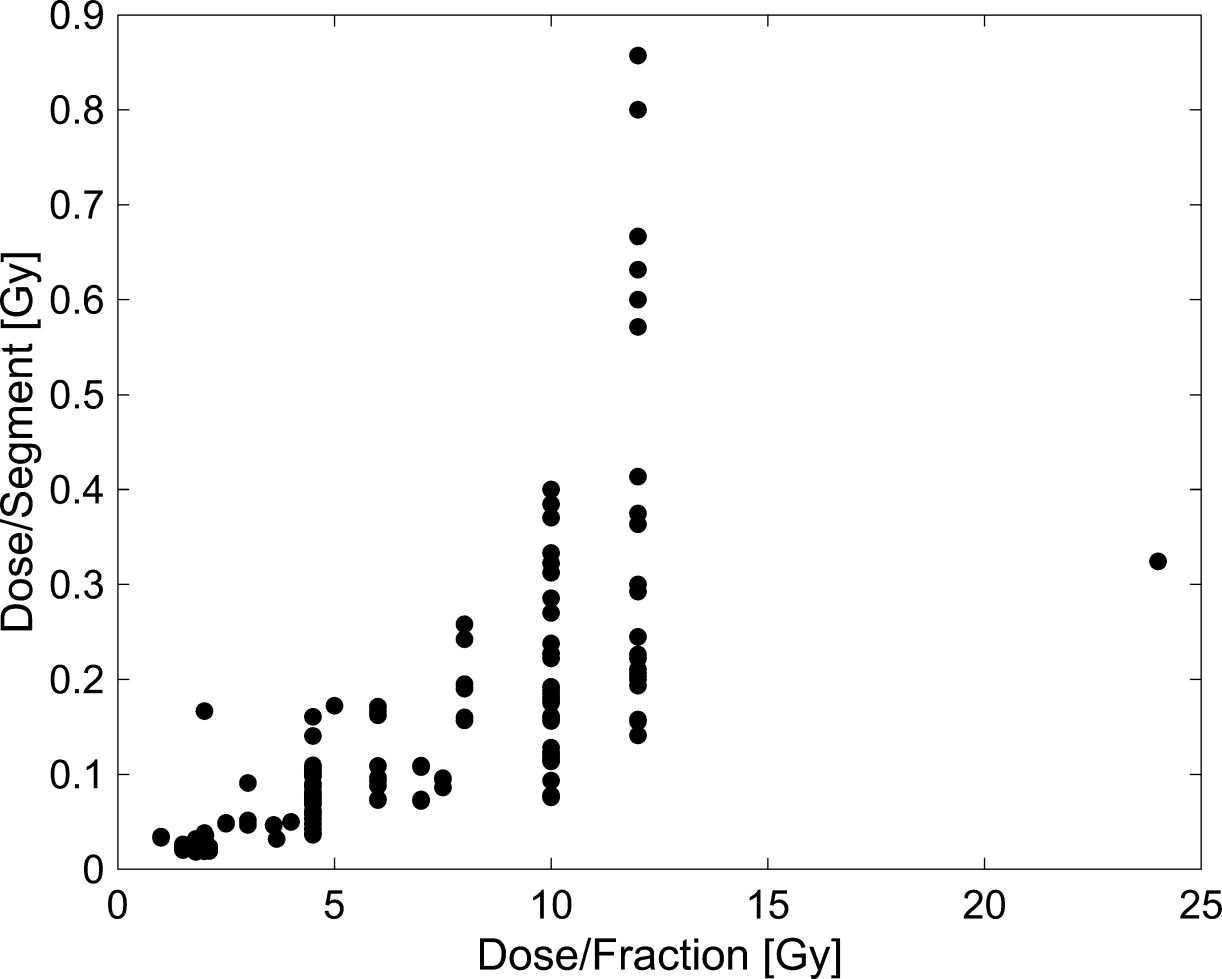
Dose per Segment vs Dose per Fraction. A general increase in the dose per segment with increasing dose per fraction was observed.

Considering the dark current contribution on the basis of the planned dose and the plan parameters resulted in an observable dependence. The dark current contribution relative to the delivered dose per segment was observed to decrease with increasing dose per segment. For dose per segment values of <0.1 Gy, 0.1‐0.2 Gy and >0.2 Gy, the dark current contribution across all fractions was 1.07% ± 0.68%, 0.42% ± 0.17%, and 0.28% ± 0.12%, respectively. The dark current percentage contribution relative to the dose per segment is shown in Fig. 3a for all fractions, and Fig. 3b for all patients. Furthermore, considering the dark current contribution relative to the prescription dose, an increase was observed with a decrease in the dose per fraction. For fractional doses of <5 Gy, 5‐10 Gy, and ≥10 Gy, the dark current contribution across all fractions was 1.06% ± 0.67%, 0.39% ± 0.18%, and 0.31% ± 0.14%, respectively. The dark current contribution with respect to the dose per fraction is shown in Fig. 4a for all fractions and Fig. 4b for all patients, respectively. While the dose per fraction dependence provides insight on the dark current contribution associated with different fractionation schemes, the result is a function of the current standard used in planning, where higher doses per fraction are associated with a higher dose per segment as well, as demonstrated in Fig. 5.

**Fig. 6:**
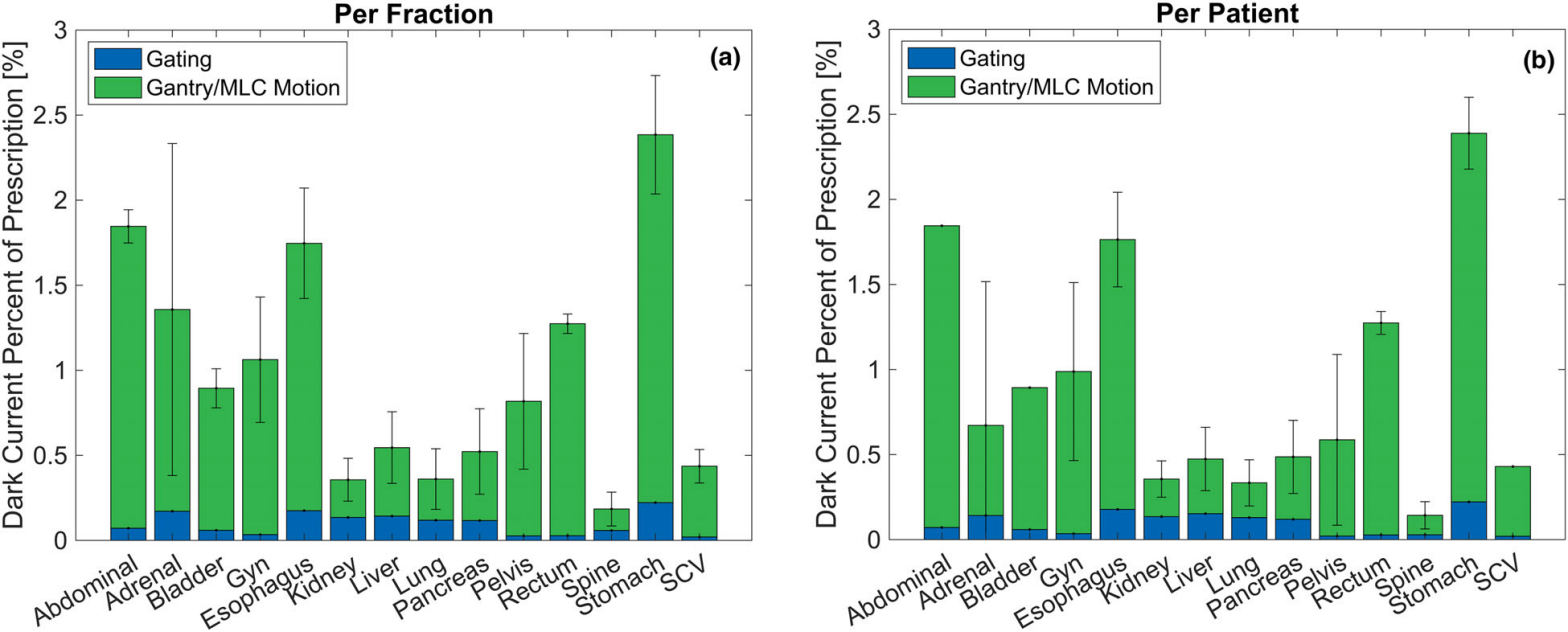
Site specific dark current contribution. The percentage contribution relative to the prescription for each site analyzed for (a) each fraction and (b) each patient. The stacked bar demonstrates the relative contribution attributable to gating and gantry/MLC motion.

Considering the impact on varying sites of treatment, the largest dark current contribution was observed for stomach (2.38% ± 0.35%) and abdominal (1.85% ± 0.10%) plans, while the smallest contribution was observed for spine (0.18% ± 0.10%), lung (0.36% ± 0.18%), and liver (0.55% ± 0.21%) plans. The larger dark current contribution for stomach and abdominal plans was characteristic of a large amount of relative time spent moving the gantry and the MLC. This was directly attributable to these plan types being associated with a low dose per segment, as they typically required a large amount of modulation to limit the dose to nearby critical structures. While a larger amount of gating is characteristic of lung and liver plans to account for motion, the low relative contribution from MLC and gantry motion limited the estimated dark current contribution. The estimated dark current contribution for each of the sites analyzed is provided in Fig. 6a for all fractions, and Fig. 6b for all patients.

## 4 DISCUSSION

The presence of a significant amount of dark current during a radiotherapy treatment has the potential to lead to a decrease in the overall quality of patient care. The methodology presented herein facilitated the monitoring of the dark current associated with the ViewRay MRIdian linac at our institution and allowed for a determination of the overall estimated impact on patient treatments. The method implemented in this work allows for independent investigation of the dark current and does not necessitate the presence a field service engineer to facilitate the measurement. There are additional methods available to the manufacturer to assess dark current contribution that would not require the use of a motion phantom, though they require the assistance of a field service engineer. The method in this work was developed to allow for an independent investigation of the dark current and allowed for the incorporation of the measurement into our monthly QA tests. Incorporating dark current measurements as part of our monthly QA allowed for frequent monitoring of the dark current rate over the first several months of machine operation and validated that the dark current was a small percentage of the beam‐on dose rate. Over the course of the first 15 months of machine operation (June 2018‐August 2019), the dark current dose rate was 0.12% ± 0.027% of the beam‐on dose rate. This value is slightly larger than the desired vendor dark current contribution of 0.1% that was established at acceptance testing. The difference between monthly measurements and values measured at installation is likely due to variability in the measurement methodology and the decreasing beam‐on dose rate observed. Our methodology compares the dark current dose rate to the beam‐on dose rate measured, and thus appreciable decreases in the dose rate over time resulted in an increased dark current contribution. That being said, since the observed beam‐on dose rate fell below the nominal dose rate at the time of acceptance (as was observed at our institution), it is reasonable to have seen a higher dark current dose rate than was originally present.

As has been mentioned, there has not been prior investigation into the dark current for MR‐linac systems to this point; however, the dark current contribution can be compared to prior works investigating a conventional linac and a CyberKnife system. Prior work by Cheng and Das reported no dark current radiation at proper machine settings for both a 6 MV and 15 MV beam on a Siemens Primus linear accelerator (0.7% for 15 MV at high IPFN values),^3^, ^4^ while Kim and Chang reported dark current contributions of up to 0.6% for a CyberKnife system.^7^ The dark current measured in this work (0.12%) demonstrated improvement compared to the 0.6% observed on the CyberKnife, though unlike the dark current quantification for the Siemens Primus, it did still have a dark current contribution present.

Utilizing the measurement of the dark current performed monthly allowed for an estimation of the dark current impact on patient deliveries. Overall, the dark current was estimated to account for 0.90% ± 0.67% of the total prescription dose. With an average estimated contribution of <1%, the dark current appeared to be sufficiently low for the majority of the treatments analyzed. However, the maximum estimated contribution for a fraction was as large as 3.61%. This not only represents an increase in the total body dose, but also a decrease in the accuracy of the plan delivery. While the analysis permitted a determination of the potential contribution of the dark current to the overall prescription dose, an understanding of this contribution on clinical outcomes warrants further investigation. This would then potentially allow for a determination of the dark current level at which the effects are expected to become clinically significant. Additionally, a limitation of this work is the consideration of aperture size when considering the effect of dark current on patient deliveries. It is not unreasonable to expect the beam apertures between segments to be smaller than the 10 × 10 cm^2^ field investigated at monthly measurements and for determination of the percentage of dose attributable to dark current radiation. Therefore, while an estimate is provided based on the quantified dark current from monthly measurements, it is likely to represent a near‐worst‐case scenario when projecting to patient deliveries. Future work could potentially investigate the use of active dosimeters during the delivery of patient treatments to more accurately characterize the true contribution from dark current.

The fractions containing larger dark current contributions warranted a further investigation of plan parameters that may be indicative of increased dark current contribution. The linac is put into the beam‐hold state between segments of the delivery and when the beam is gated due to target motion. Through an analysis of the time in each of these scenarios, it was observed that the largest estimated dark current contribution was due to motion of the gantry and the MLC, accounting for 87% of the total time spent in the beam‐hold state. This is largely the result of the necessity for the MRIdian linac to utilize a step and shoot delivery, and subsequently many segments. Utilizing many segments requires a large amount of time be spent moving the gantry and MLC into position. This effect was manifested in the analysis of the dark current contribution dependence on the dose per segment. Plans with a lower dose per segment value correspondingly had a high proportion of the time spent in the beam‐hold state due to a greater relative time being spent moving the linac to the specified segment position. This effect was demonstrated in Fig. 3, where all fractions having a dark current contribution of greater than 1% also had a dose per segment value of less than or equal to 0.1 Gy. Additionally, since the bulk of the beam‐hold time was due to gantry and MLC motion, additional motion management techniques, such as coaching, would be expected to have minimal impact on the overall dark current contribution observed. While coaching could potentially limit the amount of time spent in the beam‐hold state due to gating, our analysis showed this contribution to be minor compared to the beam‐hold time associated with motion between plan segments.

A similar dependence in the dark current contribution was observed when analyzing the dose per fraction; as the dose per fraction was decreased, the dark current contribution tended to increase. This was largely the effect of larger fraction sizes being associated with higher doses per segment as demonstrated in Fig. 5. While the dose per fraction was inherently linked to the dose per segment under the current planning standard, Fig. 4 still demonstrates the effect that a fractionation scheme may have on the expected dark current contribution. SBRT is associated with higher doses per fraction, and correspondingly larger doses per segment, leading to a decreased dark current contribution observed. Further considering the contribution of dark current with varying plan type, it was observed that abdominal and stomach plans were associated with a large dark current contribution, largely attributable to MLC/gantry motion between segments. This result is associated with a low dose per segment for these types of plans, resulting from a high level of modulation to avoid nearby critical structures. This indicates the potential for larger dark current contributions that may be present when treating sites very near critical organs that will require a larger amount of modulation. This being said, having an understanding of what the increase in dark current may be can be useful when considering the potential decrease in dose to surrounding critical structures by increasing the modulation.

The increased time spent in the beam‐hold state when utilizing many segments makes it prudent for dosimetrists and physicists to consider the impact that dark current may have on the ultimate delivery of the plan, particularly at low prescription doses. If a comparable plan quality can be achieved while utilizing a smaller number of plan segments, it may be beneficial to employ the plan with the limited number of segments as it will correspondingly decrease the dark current contribution. From our analysis, keeping the dose per segment above 0.1 Gy kept the dark current contribution below 1% in all fractions analyzed. Ultimately, the trade‐off between plan quality and plan segments must be considered on a case by case basis, but physicists should be cognizant of the dark current of their machine when implementing step‐and‐shoot deliveries.

It should also be noted that the manufacturer has incorporated recent upgrades to the system that will likely decrease the dark current contribution. Our institution has recently received both a replacement of the linac and an upgrade of the MLC. The MLC has now been updated to move at a higher speed, with leaf speeds of approximately 4.5 cm/s as opposed to the 1.5 cm/s of the MLC present in this work. Both the linac upgrade and the increased leaf speed are expected to have an impact moving forward on the dark current as investigated in this work. It is believed that the dark current rate observed relative to the beam on dose rate was largely due to the initial linac manufacturing, and thus the upgraded linac installation will lead to a decreased dark current rate. Initial investigations of the dark current dose rate post‐linac upgrade have yielded lower dark current dose rates than what was observed prior the linac upgrade. Additionally, the increase in the MLC speed will lead to a likely decrease in the overall dark current contribution towards the overall prescription. With the faster leaf speed, it is expected that less time will be spent in the beam‐hold state (as the majority of this time was MLC/gantry movement), and subsequently the dark current contribution will be decreased. Future work will investigate the potential reductions in dark current and its contribution toward the overall prescription with the most recent upgrade to the system. That being said, this work addresses our initial experience quantifying the dark current for the ViewRay MRIdian linac and would be representative of systems that have not yet received this upgrade.

## 5 CONCLUSION

Overall, the relative dark current dose rate compared to the beam‐on dose rate was investigated for the ViewRay MRIdian linac at our institution with monthly values (June 2018‐August 2019) being observed slightly above the acceptance testing limits put forth by the manufacturer. The overall dark current contribution was associated with the beam‐on dose rate of the machine at the time of measurement, and a decrease from the nominal dose rate resulted in an increased relative dark current dose rate. Determination of the potential contributions of the observed dark current on patient deliveries was analyzed, revealing dark current contributions that on average appeared to be at an acceptable level relative to the prescription dose, though the true impact on clinical outcomes warrants further investigations. Deliveries that had a large overall dark current contribution were associated with a small dose per treatment segment. It is recommended that, to the extent possible, the dose per segment be maximized while maintaining a comparable plan quality in order to limit the potential impact of dark current. With new linac technology implementing step‐and‐shoot deliveries as opposed to arc deliveries, it would be judicious of users to perform measurement of the dark current after linac installation, and potentially on a regular basis to verify limited contribution. As dark current has not been addressed in the literature in recent years, this work serves as a reminder that even as technology continues to advance, a return to prior methods and considerations may be necessary for optimal clinical implementation.

## CONFLICT OF INTEREST

Dr. Mittauer reports personal fees from ViewRay Inc., during the conduct of the study; Dr. Bayouth reports membership of Advisory Board of ViewRay Inc., during the conduct of the study.
